# The reduction of birth weight by fine particulate matter and its modification by maternal and neighbourhood-level factors: a multilevel analysis in British Columbia, Canada

**DOI:** 10.1186/s12940-016-0133-0

**Published:** 2016-04-14

**Authors:** Anders C. Erickson, Aleck Ostry, Laurie H. M. Chan, Laura Arbour

**Affiliations:** Division of Medical Sciences, University of Victoria, Medical Science Bld. Rm-104, PO Box 1700 STN CSC, Victoria, V8W 2Y2 BC Canada; Department of Geography, University of Victoria, David Turpin Bldg. Rm-B203, PO Box 1700 STN CSC, Victoria, V8W 2Y2 BC Canada; Center for Advanced Research in Environmental Genomics, University of Ottawa, 20 Marie-Curie, Ottawa, K1N 6N5 ON Canada; Department of Medical Genetics, University of British Columbia, C201 - 4500 Oak Street, Vancouver, V6H 3N1 BC Canada

**Keywords:** Birth weight, Air pollution, Multilevel model, Neighbourhood effects, Particulate matter, Socioeconomic status, Effect modification

## Abstract

**Background:**

The purpose of this research was to determine the relationship between modeled particulate matter (PM_2.5_) exposure and birth weight, including the potential modification by maternal risk factors and indicators of socioeconomic status (SES).

**Methods:**

Birth records from 2001 to 2006 (*N* = 231,929) were linked to modeled PM_2.5_ data from a national land-use regression model along with neighbourhood-level SES and socio-demographic data using 6-digit residential postal codes. Multilevel random coefficient models were used to estimate the effects of PM_2.5_, SES and other individual and neighbourhood-level covariates on continuous birth weight and test interactions. Gestational age was modeled with a random slope to assess potential neighbourhood-level differences of its effect on birth weight and whether any between-neighbourhood variability can be explained by cross-level interactions.

**Results:**

Models adjusted for individual and neighbourhood-level covariates showed a significant non-linear negative association between PM_2.5_ and birth weight explaining 8.5 % of the between-neighbourhood differences in mean birth weight. A significant interaction between SES and PM_2.5_ was observed, revealing a more pronounced negative effect of PM_2.5_ on birth weight in lower SES neighbourhoods. Further positive and negative modification of the PM_2.5_ effect was observed with maternal smoking, maternal age, gestational diabetes, and suspected maternal drug or alcohol use. The random intercept variance indicating between-neighbourhood birth weight differences was reduced by 75 % in the final model, while the random slope variance for between-neighbourhood gestational age effects remained virtually unchanged.

**Conclusion:**

We provide evidence that neighbourhood-level SES variables and PM_2.5_ have both independent and interacting associations with birth weight, and together account for 49 % of the between-neighbourhood differences in birth weight. Evidence of effect modification of PM_2.5_ on birth weight across various maternal and neighbourhood-level factors suggests that certain sub-populations may be more or less vulnerable to relatively low doses PM_2.5_ exposure.

## Background

Studies of exposure to particulate air pollution have consistently shown an association with low birth weight, a predictor of fetal growth restriction and important determinant of infant and child-wellbeing [1–3]. The fine fraction of particulate matter (PM_2.5_ - less than 2.5 μm) is a complex mixture of elemental and organic carbon compounds, metals and gases that stem predominantly from vehicle exhaust, residential heating and industrial emissions. PM_2.5_, which includes ultrafine particles less than 0.1 μm, can penetrate deep into the pulmonary alveolar tissue where inflammatory mediators and possibly the particles themselves translocate into the bloodstream causing systemic cardiovascular and immunological alterations such as platelet activation, coagulation and endothelial dysfunction [4–6]. These physiological changes extend to the placenta, a highly vascularized organ and extension of the maternal cardiovascular system with similarly affected endothelial cellular tissues particularly susceptible to oxidative and inflammatory injury [7–9]. Excess or uncontrolled oxidative stress and inflammation early in pregnancy may disrupt placental cell growth and differentiation, potentially leading to deficient deep placentation and morphological adaptations associated with several adverse pregnancy outcomes including fetal growth restriction [10].

These mechanisms by which PM_2.5_ may act to adversely impact the reproductive system are not fully understood; however, evidence supports the potential for a shared mode of developmental toxicity with several other known risk factors [5, 11]. This includes factors that also promote or are associated with oxidative stress and inflammation such as smoking [12], drug use [13], advanced maternal age [14] gestational diabetes [15], and low socioeconomic status (SES) in general [16]. The causal pathways in which SES contributes to adverse pregnancy outcomes can be conceptualized in terms of ‘downstream’ or mediating exposures, stresses and behaviours acting on the individual through ‘upstream’ society-level determinants such as poverty, poor education, income inequality and social discrimination/marginalization over the lifespan [17, 18]. This impact of the social environment on health behaviours and outcomes creates hierarchical structures within which individuals are nested in neighbourhoods and communities with their own set of attributes that can promote or antagonize health and healthy behaviours [19, 20].

A particular challenge in environmental epidemiology is handling data at differing geographic scales. Birth registries and vital statistics provide data on individual births and certain risk factors, but may not have data on socially patterned risk factors. Alternatively, reliable SES data such as education, income and housing quality are often only available from national census databases using arbitrary administrative spatial units. Finally, obtaining individual-level environmental exposure data is often not possible. Therefore, the epidemiologist is often left with a mix of individual-level observations clustered within neighbourhood areas each with distinct attributes. The use of multilevel statistical models separates the individual-level effects from the context of their social and physical environments and can therefore quantify the degree of clustering of individuals within neighbourhood areas and test whether neighbourhood factors themselves have direct effects on the health outcome or act indirectly via the modification of individual-level variables [21, 22].

Through the mechanisms of oxidative stress and inflammation there is evidence that SES may not only confound but modify the PM_2.5_-birth outcome relationship [23–25]. Various exposures and experiences may act in a non-additive manner to influence fetal development [5]. We present a multilevel cross-sectional analysis of the association between birth weight and PM_2.5_ in British Columbia, Canada where levels of PM_2.5_ are relatively low but can vary substantially between different communities [26]. We explore the potential for between-neighbourhood variability for the slope of gestational age on birth weight and whether interactions with PM_2.5_, neighbourhood-level SES indicators, and/or individual-level risk factors are able to explain any neighbourhood-level variability. We had three research questions: 1) does exposure to PM_2.5_ and residence in low SES neighbourhoods in BC have significant independent negative associations with birth weight? 2) does the effect of gestational age on birth weight differ between neighbourhoods? 3) does PM_2.5_ interact with neighbourhood-level SES and/or individual-level risk factors to modify their independent effects on birth weight to help explain any neighbourhood-level differences?

## Methods

This was a population-based retrospective cohort of singleton births in British Columbia from 2001 to 2006 (*N* = 237,470). Data from the BC Perinatal Data Registry were provided by Perinatal Services British Columbia (PSBC) which included information on maternal-infant health status and outcomes, reproductive history, maternal risk factors and attributes, and residential postal codes. The Registry accounts for 99 % of births and stillbirths in BC of at least 20 weeks gestation or at least 500 g birth weight. Research data access is provided by a Partnership Accord /Memorandum of Agreement between all BC Health Authorities and PSBC through the *Freedom of Information and Privacy Protection Act* [27]. Research ethics board approval was granted by the University of Victoria (ethics protocol #: 11-043).

The outcome variable was continuous birth weight of singleton births. In order to avoid potential selection bias, we included all births (stillbirth and live) for all gestational ages (20–44 weeks). Excluded records included out-of-province and invalid postal codes (*n* = 1096), non-viable births prior to 20 weeks gestation or < 500 g (*n* = 14), and the list-wise deletion of births missing important data including: cigarettes smoked per day (cigs/day, *n* = 2501), PM_2.5_ (*n* = 1510), gestational age (*n* = 373), and birth weight (*n* = 41). All continuous variables, except cigarettes/day, were standardized and centred to ease interpretation and aid model convergence.

The spatial location of each birth record was geocoded based on the latitude-longitude coordinate of the mother’s residential postal code at the time of delivery using *GeoRef* [28]. Birth records were related to their corresponding census dissemination area (DA) by performing a point-in-polygon spatial join procedure in *ArcGIS 10.2* [29]. DAs are the smallest geographical unit for which census data are available and represent neighbourhood blocks ranging between 200 and 800 people. While DAs do not necessarily represent existing neighbourhood communities [30], they can act as proxies for a general catchment area of personal home-life activities [31, 32]. Birth records were identified as being either rural or urban using the Statistics Canada Metropolitan Influence Zone (MIZ) codes which are based on commuting flows of small towns into larger cities and metropolitan areas [33].

Exposure to PM_2.5_ was estimated using a national land-use regression (LUR) model developed to estimate PM_2.5_ at the census street block-face level [34]. The model used a number of predictors including satellite measures, proximity to major roads and industry to account for 46 % of the variability in measured annual PM_2.5_ concentrations. Unlike nitrogen dioxide (NO_2_), PM_2.5_ tends to have a more homogeneous intra-urban distribution between personal, indoor and ambient exposure [35]. The LUR model estimates used for this study showed very little variability of PM_2.5_ exposures between individuals within a given DA. We therefore aggregated the point-level estimates of PM_2.5_ to their DA-level mean and related it to individual birth records as an area-level variable.

The DA-level SES and demographic data were represented by three related but independent datasets all based on the 2006 Statistics Canada national census. The first was a Canadian SES index (SESi) developed by Chan et al. [36]. The second was an education variable representing the proportion of population over 15 with any post-secondary education, including college, trades, or university. The third was the proportion of continental Asian immigrants by DA as it has been shown in BC and elsewhere that healthy babies from Asian and South Asian backgrounds are constitutionally smaller compared to their Caucasian counterparts [37, 38]. Asian and South Asian ethnicities are well-represented throughout BC but particularly in concentrated pockets throughout the major urban center of Metro Vancouver where levels of PM_2.5_ are also high. The correlation between immigrant density with SESi and PM_2.5_ was −0.62 and 0.53 respectively (*p* < 0.001); we therefore created a residual immigrant density variable using a sequential regression technique [39]. Here, immigrant density was regressed against SESi and PM_2.5_ with the saved residuals representing the uncorrelated and independent contribution of immigrant density on birth weight freed from its collinearity with SESi and PM_2.5_. This same method was used between SESi and education (r = 0.25) creating a residual education variable. The education and immigrant data were obtained by access to ABACUS via the Data Liberation Initiative [40].

In order to avoid data loss from rural DAs, imputation for missing SES, education and immigrant density values was performed. Taking advantage of the nested hierarchical structure of the administrative census and health boundaries, the mean value for a larger encompassing census subdivision (CSD) or local health area (LHA) was imputed for a nested DA with a missing value. There were 1441 values imputed in 52 DAs for SESi (0.6 % of final N, 0.8 % of DAs), and 3170 values imputed in 108 DAs for both education and immigrant density (1.4 % of final N, 1.7 % of DAs). Sensitivity analyses were performed using only the non-missing data.

Hierarchical (multilevel) linear regression models were used to test our research questions, thereby accounting for the clustering, or non-independence, of individuals (level-1) belonging to a given DA neighbourhood (level-2). The multilevel model allows the intercept and slope to act as random parameters having between-area (DA) variability from an overall (BC-wide) mean intercept and slope. That is, each DA has its own intercept and slope in which their variability from the overall intercept and slope can be investigated with the addition of level-1 and level-2 variables and their interactions [41]. We followed a bottom-up approach to model building to quantify the explained proportional change in variance (PCV), the multilevel model equivalent to an *R*^*2*^ [22]. We started with an empty (null) random intercept model without any independent variables in which birth weight is only a function of the mother’s residential DA. The presence of significant random intercept variance indicates there are unexplained between-neighbourhood differences in mean birth weight. The proportion of the total variance in birth weight that arises due to neighbourhood differences can be quantified by computing the intra-class correlation (ICC), and hence provides the degree of clustering of individual birth weight within neighbourhoods [22].

Gestational age was added to the null model and given a random slope (i.e., the mean within-DA effect of gestational age on birth weight was allowed to differ between DAs). The presence of a significant random slope indicates that its effect is not constant (or equal) for all DAs. Subsequent models included the individual and DA-level variables along with cross-level and within-level interactions in order to assess their fixed effects on birth weight but to also determine if their inclusion addressed any unexplained intercept or slope variance. Models were tested using the Akaike Information Criterion (AIC) to evaluate model performance. All statistical analyses were conducted in *Stata 13IC* [42].

Finally, while multilevel models address *intra*-area dependence while quantifying *inter*-area variance, they assume spatial independence among neighbouring areas. However environmental and social processes can extend beyond arbitrary neighbourhood boundaries. Additionally as mentioned above, census DAs do not necessarily represent neighbourhood dynamics, services, infrastructure, etc.; and evidence of spatial clustering between DAs may indicate that an alternative neighbourhood areal unit should be considered. We used spatial methods to test for this by checking the level-1 model residuals and level-2 predicted random parameters (intercepts and slopes) for spatial autocorrelation using the local Moran’s I statistic [43]. The presence of significant residual spatial autocorrelation indicates the existence of unobserved spatial processes causing DAs to cluster and can be a sign of model misspecification. Prediction of the DA-level random intercept and slope errors used an Empirical Bayes method [44] available as a post-estimation command in *Stata 13IC*.

## Results

After exclusions there were 231,929 singleton (live and stillborn) births located in 6338 neighbourhood DAs (min. = 1, max. = 781, avg. = 37). Table 1 summarizes the untransformed individual and neighbourhood covariates (non-centered, non-standardized) along with their relationship to PM_2.5_. Table 2 reports the adjusted coefficients for the individual and DA-level covariate fixed effects on continuous birth weight (Model 1 to 3). Gestational age was modeled using a quadratic term to account for the rapid fetal growth in mid-gestations and its slower growth post-term (>36 weeks). Maternal smoking (cigarettes/day) and PM_2.5_ were also modeled using a quadratic terms, both indicating a subdued dose–response with increasing exposure.

**Table 1:** Descriptive statistics^a^ for individual (Level-1) and DA (Level-2) covariates

Variables	Mean	Std. Dev.	Min-Max	PM 2.5 mean (SE)^b^	
				Absence/1st quintile	Presence/5th quintile
Level-1 (individual)					
Birth weight (grams)	3433.3	566.51	135 – 6475	7.30 (.016)	7.36 (.018)^c,e^
Gestational age (weeks)	38.8	2.02	19 – 44	7.30 (.016)	7.30 (.017)^f^
Maternal age (years)	29.8	5.60	11 – 55	7.10 (.022)	7.39 (.014)^c^
Nulliparous	0.45	0.50	0 – 1	7.27 (.016)	7.34 (.016)^c^
Gestational diabetes	0.06	0.25	0 – 1	7.29 (.016)	7.53 (.016)^c^
Pre-existing diabetes	0.004	0.06	0 – 1	7.30 (.016)	7.34 (.034)
Gestational hypertension	0.02	0.15	0 – 1	7.30 (.016)	7.44 (.019)^c^
Poor prenatal care	0.09	0.29	0 – 1	7.28 (.016)	7.47 (.02)^c^
Drug/Alcohol flag	0.02	0.15	0 – 1	7.31 (.016)	7.08 (.026)^c^
Cigarettes/day	0.79	2.91	0 – 20	7.33 (.015)	7.02 (.023)^c,g^
Fall/Winter season	0.48	0.50	0 – 1	7.29 (.016)	7.31 (.016)^c^
Level-2 (DA) variables					
SESi	-0.08	0.58	-2.22 – 1.18	7.82 (.027)	6.95 (.032)^c,d^
Higher education	0.50	0.12	0 – 0.95	7.16 (.04)	7.53 (.022)^c,d^
Immigrant density	0.16	0.19	0 – 0.86	6.75 (.023)	7.95 (.021)^c,d^
Rural address	0.11	0.32	0 – 1	7.39 (.014)	6.59 (.061)^c,d^
PM_2.5_ (μg/m^3^)	7.30	0.86	4.41 – 10.23	--	--

^a^Values shown are unstandardized, non-centered; Birth weights below 500 grams were included if their gestational age was > 19 weeks. Gestational age under 20 weeks were included if their birth weight was >499 grams. Poor prenatal care: having less than 4 prenatal care visits or was missing; Drug/Alcohol flag: indicates whether physician lists patient’s use of alcohol or drugs (prescription, nonprescription, illicit) as a risk factor in this pregnancy; Cigarettes/day: self-reported number of cigarettes smoked daily at 1st prenatal visit (excluding non-smokers, mean(sd) = 7.7 (5.41)); Fall/Winter season: birth month = September to February

^b^Robust standard errors adjusted for 6338 DA clusters

^c^Significant difference at *p* < 0.05 using Wald tests

^d^1st vs. 5th quintile

^e^Normal birth weight vs. low birth weight

^f^Term birth vs. preterm birth

^g^Non-smoker vs. current smoker

**Table 2:** Adjusted individual and DA-level fixed effects on continuous birth weight

Variables	Model-1	Model-2	Model-3
	β (95 % CI)	β (95 % CI)	β (95 % CI)
Level-1 (individual)			
Gestational age	310.2 (307.6 – 312.7)	308.7 (306.1 – 311.2)	308.5 (306.0 – 311.1)
Gestational age^a^	-11.6 (-12.2 – -11.1)	-11.9 (-12.4 – -11.3)	-11.9 (-12.5 – -11.4)
Maternal age	-6.6 (-8.6 – -4.7)	-6.0 (-8.0 – -4.0)	-4.7 (-6.6 – -2.7)
Nulliparous	-137.2 (-141.0 – -133.4)	-135.7 (-139.5 – -131.9)	-134.8 (-138.6 – -131.1)
Gestational diabetes	54.5 (47.1 – 61.8)	60.0 (52.7 – 67.4)	62.0 (54.6 – 69.4)
Pre-existing diabetes	320.7 (292.1 – 349.3)	320.6 (292.1 – 349.1)	321.2 (292.6 – 349.7)
Gestational hypertension	-90.1 (-102.3 – -77.9)	-88.9 (-101.0 – -76.7)	-87.6 (-99.7 – -75.4)
Prenatal care visits	-59.0 (-65.3 – -52.8)	-55.0 (-61.2 – -48.7)	-52.2 (-58.4 – -45.9)
Drug/Alcohol flag	-79.1 (-91.2 – -67.1)	-79.2 (-91.2 – -67.2)	-81.7 (-93.7 – -69.7)
Cigarettes/day	-20.8 (-22.5 – -19.0)	-22.0 (-23.8 – -20.3)	-22.7 (-24.4 – -20.9)
Cigarettes/day^a^	0.63 (0.51 – 0.74)	0.68 (0.57 – 0.79)	0.7 (0.59 – 0.82)
Fall/Winter season	−−	−−	-6.8 (-10.4 – -3.2)
Level-2 (DA)			
SESi	−−	37.4 (35.2 – 39.7)	29.4 (27.0 – 31.8)
Higher education	−−	-2.1 (-4.4 – 0.2)	3.0 (0.7 – 5.3)
Immigrant density	−−	-29.2 (-31.4 – -26.9)	-31.3 (-33.5 – -29.1)
Rural address	−−	4.8 (-3.4 – 12.9)	-14.6 (-22.6 – -6.7)
PM_2.5_	−−	−−	-23.9 (-26.5 – -21.3)
PM_2.5_^a^	−−	−−	2.8 (1.3 – 4.3)

See Table 1 legend for variable definitions; ^a^Variables were modeled as quadratics

**Fig. 1:**
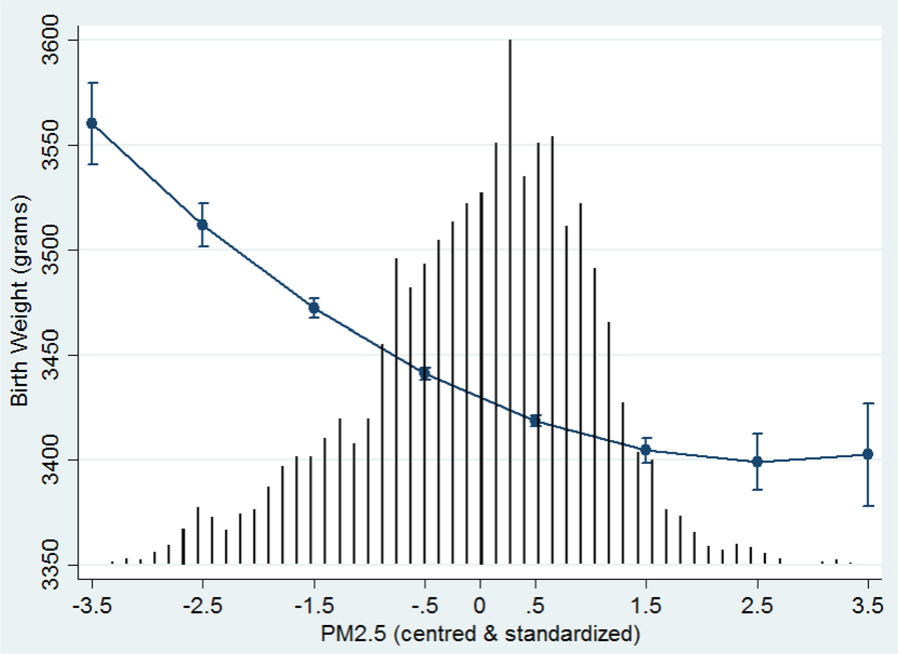
Adjusted predicted effects of PM_2.5_ on birth weight. Predicted effects of PM_2.5_ on birth weight with 95 % confidence intervals are conditional on model covariates included in Model 4. Black vertical lines represent the frequency distribution of PM_2.5_

Model 2 added the DA-level variables of SESi, education, immigrant density and rural residence. Their fixed effects show that lower SES and higher Asian immigrant density were significantly associated with lower birth weights (Table 2). Rural DAs and DAs with higher proportion of post-secondary education were not significantly associated with birth weight in this model. However, both became significant after the addition of PM_2.5_ and season of birth (cold vs. warm) in Model 3. Higher education had a positive association with birth weight, while rural areas had a significant negative association with birth weight. PM_2.5_ was found to have a significant non-linear negative association on birth weight whereby the negative effect tapers off at higher concentrations of PM_2.5_ (Fig. 1). Being born in a cold (fall or winter) month also had a significant negative association with birth weight (Table 2).

**Fig. 2:**
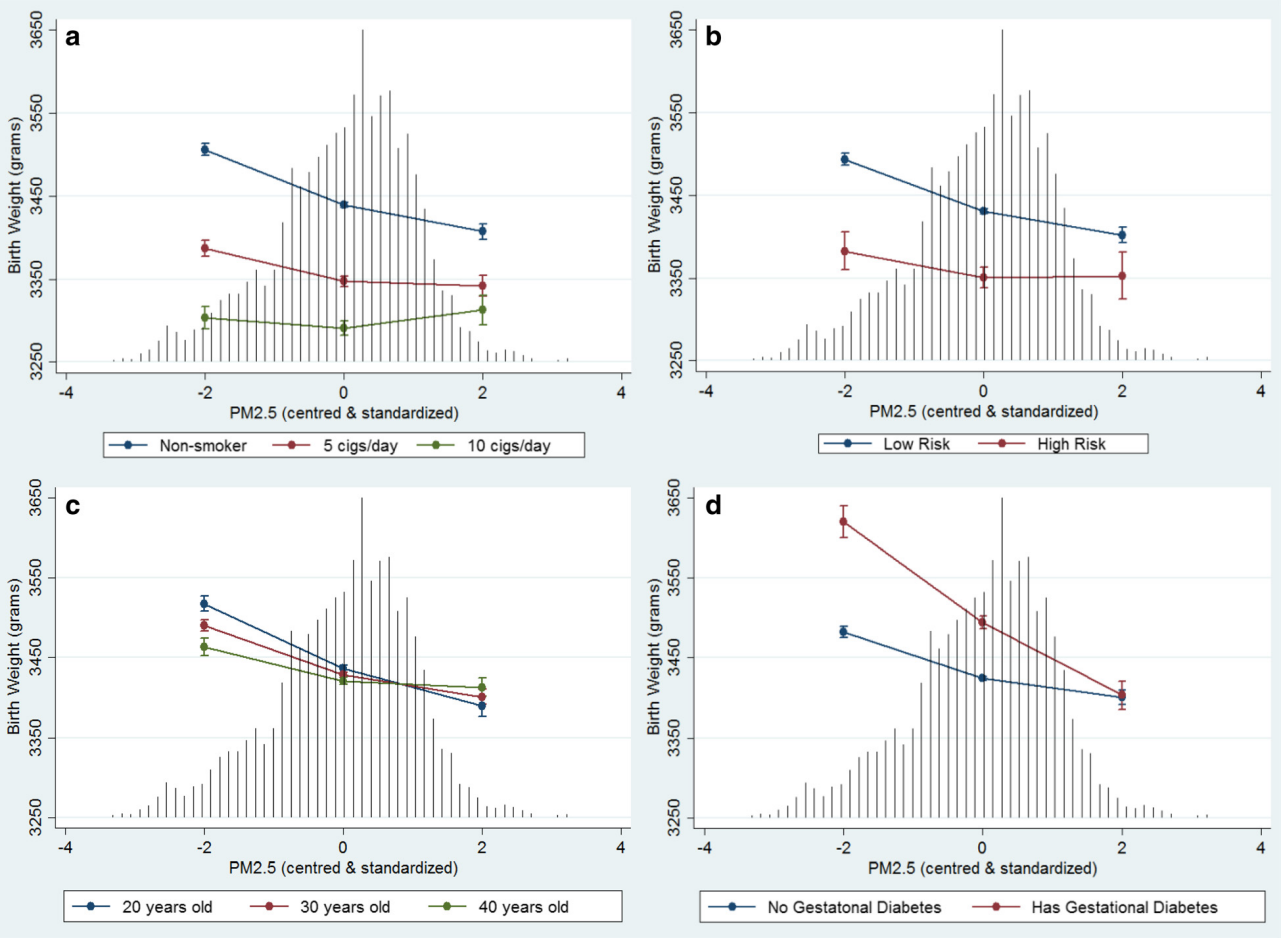
Adjusted predicted effects of maternal risk factors on birth weight across levels of PM_2.5_. **a** Maternal Smoking **b** Suspected Drug or Alcohol Use **c** Maternal Age **d** Gestational Diabetes. Predicted effects on birth weight with 95 % confidence intervals are conditional on model covariates included in Model 4. Black vertical lines represent the frequency distribution of PM_2.5_

**Fig. 3:**
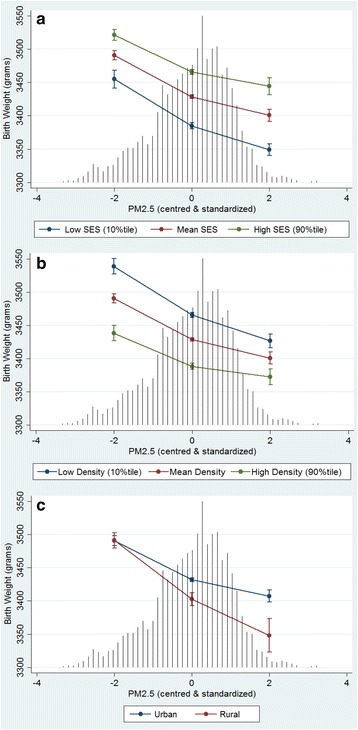
Adjusted predicted effects of DA-level factors on birth weight across levels of PM_2.5_. **a** Socioeconomic Status Index (SESi) **b** Asian Immigrant Density **c** Rural Residence. Predicted effects on birth weight with 95 % confidence intervals are conditional on model covariates included in Model 4. Black vertical lines represent the frequency distribution of PM_2.5_

Model 4 tested interactions with PM_2.5_ including cross-level (level-1 by level2) and level-2 by level-2 interactions to explain the between-DA random intercept variability. The model results are presented in Tables 3 and 4 including the main effects as well as the interaction effects with PM_2.5_. Four maternal variables showed effect modification with PM_2.5_ on birth weight. Maternal smoking and suspected drug or alcohol use both had positive interactions with PM_2.5_ on birth weight revealing a subdued association with increased PM_2.5_ exposure (Fig. 2a and b respectively). Maternal age was also modified by differences in PM_2.5_ exposure with younger maternal ages showing a larger reduction in birth weight with increased PM_2.5_ exposure (Fig. 2c). Alternatively, gestational diabetes was associated with a much greater reduction in birth weight with increasing PM_2.5_ compared to normal births, essentially nullifying the higher birth weights produced by the condition (Fig. 2d). Three DA-level variables showed significant effect modification with PM_2.5_ on birth weight. First, the interaction between SESi and PM_2.5_ revealed a more pronounced effect of PM_2.5_ in lower SES neighbourhoods (Fig. 3a). Higher Asian immigrant density buffered the PM_2.5_ effect (Fig. 3b), while rural DAs showed an additional reduction in birth weight with increasing PM_2.5_ levels compared to urban DAs (Fig. 3c).

**Table 3:** Adjusted individual and DA-level fixed effects on continuous and term birth weight and their modification by PM_2.5_ (Model-4)

Variables	Main effect	Modification by PM2.5	Corresponding figure
	β (95 % CI)	β (95 % CI)	
PM_2.5_^a^	-22.4 (-25.2 – -19.7)	4.9 (3.2 – 6.7)	1
Cigarettes/day^a^	-22.0 (-23.8 – -20.2)	2.8 (2.2 – 3.4)	2A
Drug/Alcohol flag	-80.6 (-93.0 – -68.2)	15.3 (4.4 – 26.2)	2B
Maternal age	-4.2 (-6.2 – -2.2)	5.5 (3.7 – 7.4)	2C
Gestational diabetes	70.2 (62.6 – 77.8)	-33.8 (-41.9 – -25.8)	2D
SESi	30.2 (27.7 – 32.7)	4.6 (2.0 – 7.2)	3A
Immigrant density	-33.3 (-35.8 – -30.7)	6.3 (3.3 – 9.2)	3B
Rural address	-29.1 (-39.1 – -19.1)	-16.4 (-24.5 – -8.3)	3C

^a^Modeled as a quadratic, Cigarettes/day quadratic term: 0.7(0.6 – 0.8); Model adjusted for gestational age, nulliparous, diabetes mellitus, gestational hypertension, prenatal care visits, season of birth, DA-level education

**Table 4:** Sensitivity analysis using only term birth and excluding stillbirths and congenital anomalies (*N* = 207,405)

Variables	Main effect	Modification by PM2.5
	β (95 % CI)	β (95 % CI)
PM_2.5_^a^	-24.1 (-26.9 – -21.2)	5.0 (3.2 – 6.8)
Cigarettes/day^a^	-21.9 (-23.8 – -20.0)	2.8 (2.2 – 3.4)
Drug/Alcohol flag	-80.1 (-93.6 – -66.6)	13.2 (1.2 – 25.2)
Maternal age	-3.1 (-5.2 – -1.1)	5.0 (3.1 – 6.9)
Gestational diabetes	60.2 (52.1 – 68.4)	-30.7 (-39.2 – -22.1)
SESi	30.5 (27.9 – 33.1)	4.3 (1.5 – 7.0)
Immigrant density	-36.0 (-38.7 – -33.3)	6.9 (3.8 – 9.9)
Rural address	-31.2 (-41.6 – -20.8)	-16.9 (-25.3 – -8.5)

^a^Modeled as a quadratic, Cigarettes/day quadratic term: 0.7(0.6 – 0.8); Model adjusted for gestational age, nulliparous, diabetes mellitus, gestational hypertension, prenatal care visits, season of birth, DA-level education

The random effects, the explained proportional change in variance (PVC), and model diagnostics are presented in Table 5. The unadjusted ICC for the Null random-intercept model was 0.019, indicating that 1.9 % of the total residual differences in birth weight are attributable to DA-level contextual factors. The inclusion of the level-1 covariates along with the random slope for gestational age, the ICC_adj_ (now conditional on mean-centred gestational age, 38.8 weeks), increased to 2.3 %. This was due to the large reduction in the level-1 residual variance (560.5 to 435.2) relative to the reduction in the level-2 random intercept variance (78.8 to 67.0). The addition of DA-level variables in Model 2 and Model 3 removed a lot of the DA-level variance reducing the ICC_adj_ to 1.1 and 0.8 % respectively.

**Table 5:** Random effects and model diagnostics from hierarchical linear models for continuous birth weight in BC, Canada

Random effects & model diagnostics	Null model	Null + r.slope	Model-1 (level-1)	Model-2 (SES)	Model-3 (PM_2.5_)	Model-4 (PM_2.5_ interact)
Variance components						
L1 residual (sd)	560.5	442.9	435.2	435.3	435.3	435.1
L2 intercept (sd)	78.8	67.6	67.0	45.9	39.7	37.9
L2 slope (sd)	--	30.1	26.6	27.0	27.2	27.4
Intercept	3434.2	3448.4	3524.2	3521.3	3523.4	3522.6
AIC	597007	489148	480867	479403	478997	478753
L1-PCV (%)	Ref	37.5	39.7	39.7	39.7	39.7
L2-PCV (%)	Ref	26.4	27.7	66.0	74.5	76.8
ICC/VPC^a^	0.019	0.023^a^	0.023^a^	0.011^a^	0.008^a^	0.008^a^
L1 Moran’s I^b^	0.122	0.108	0.105	0.043	0.022	0.018
L2ri Moran’s I^b^	0.300	0.301	0.310	0.113	0.079	0.071
L2rs Moran’s I^b^	--	0.018	0.018	0.018	0.017	0.017

L1: level-1 = individual-level; L2: level-2 = DA-level; sd: standard deviation; AIC: Akaike Information Criterion; PCV: proportional change in variance; ^a^ICC: Intra-class correlation – is called the VPC (variance partition coefficient) when conditional on the random-slope variable, thus values in table represent intercepts for individuals with mean gestational age (~39 weeks); L2ri: level-2 random intercept; L2rs: level-2 random slope; ^b^all results were significant *p* < 0.05 with 999 permutations using a queen criterion spatial weight matrix

The level-2 random intercept standard deviation indicates that the mean birth weight for every DA has a degree of variability from the overall (BC-wide) mean birth weight. For the Null model, the overall birth weight intercept is was 3434.2 g with a standard deviation of 78.8. This equals a 8.6 % difference in range between 95 % of the DAs (3434.2 ± (1.96 × 78.8) = 3280.0 and 3588.8 g). Similarly, we calculate the between-DA 95 % distributional range of slopes for gestational age to fall between 255.8 and 361.6 g (308.7 ± (1.96 × 26.6)), a 29.3 % difference in how one week gestation increases birth weight between DAs.

The level-1 and level-2 explained PCV (L1-PCV & L2-PCV) summarizes the relative degree of explained variance at the different levels between the different models. Using the Null model as the reference, the L1-model resulted in an L1-PCV & L2-PCV of 39.7 and 27.7 % respectively (Table 5). These are fairly large PCVs, indicating the within and between-DA variance in birth weight shown in the Null model was moderately attributable to these individual (level-1) compositional factors, largely gestational age. The addition of the DA-level variables in Model 2 explained an additional 38.3 % of the DA-level variance (cumulative L2-PVC = 66 %). The additions of PM_2.5_ and season of birth in Model 3 further explained an additional 8.5 % of the level-2 intercept variance beyond that of Model 2. Model 4 accounted for an additional 2.3 % L2-PCV.

Spatial analyses were used as a model diagnostic to test for significant spatial autocorrelation of the model residuals. The local Moran’s I statistics reported in Table 5 indicate the degree of local spatial autocorrelation (i.e., spatial clustering) of the level-1 (L1) residuals as well as the level-2 predicted random intercepts (L2ri) and slopes (L2rs) for all five models. Interpreted in a similar manner as a regular correlation coefficient, the Moran’s I statistic reveals the existence of significant localized clustering of residuals at both level-1 and level-2 in the Null and level-1 model. The addition of level-2 variables reduced the Moran’s I substantially; however small but significant clustering remained.

Sensitivity analyses using only the non-imputed DAs (N_1_ = 228,765 in 6230 DAs) showed very minor differences in magnitude of significant variables in the birth weight models. In a second sensitivity analysis, we restricted the sample to only term births excluding stillbirths and congenital anomalies. As expected, there was a large reduction in the random-slope variance for gestational age due to dropping preterm births but no other differences in the observed relationships (Table 4).

Finally, a check for potential collider bias was performed by omitting gestational age as a covariate from the models [45]. Random-intercept models equivalent to those presented in Tables 2, 3 and 4 were run and assessed for differences. Many of the individual-level covariates returned to resemble their unadjusted estimates listed in Table 1. The interaction between PM_2.5_ and cigarettes/day remained unchanged, whereas the PM_2.5_ interaction with drug or alcohol flag was no longer significant (*p* = 0.066). The interaction between gestational diabetes and PM_2.5_ was reduced by half, but was still significant. The effects of the DA-level variables SESi and immigrant density increased moderately beyond their 95 % CIs listed for Model 4. The effect for PM_2.5_ decreased but was still significant (-18.0 (95 % CI -21.3 – -14.7)). The DA-level interaction between PM_2.5_ and immigrant density was reduced by half and marginally not significant (*p* = 0.051), while the interaction between SESi and PM_2.5_ was also reduced to non-significance (*p* = 0.33).

## Discussion

This study employed multilevel random coefficient models to assess the effect of PM_2.5_ on birth weight and test its interaction with individual and neighbourhood-level risk factors. Our results show that individual and neighbourhood-level factors are capable of modifying the association between PM_2.5_ exposure and fetal growth. Furthermore, through the use of random-slopes models we show that the effect of gestational age on birth weight can vary considerably between neighbourhood DAs which was only moderately addressed in our models. After adjusting for individual-level covariates and DA-level socio-economic and socio-demographic variables, we found a significant non-linear effect between PM_2.5_ and birth weight in 231,929 births in British Columbia, Canada. This association was robust to the exclusion of stillbirths and congenital anomalies as well as the use of only term births and models dropping gestational age as a covariate, demonstrating that selection bias does not affect the observed PM_2.5_ main effects.

Our results corroborate the growing literature supporting a negative association between PM_2.5_ and birth weight [3, 46, 47]. Even in settings of relatively low air pollution exposure similar to our study, significant reductions in birth weight have been observed [48]. This strengthens the evidence of the low-dose effects of PM_2.5_ and is exemplified by Fig. 1 which shows the largest potential effects on birth weight are seen at the low to mid concentrations of PM_2.5,_ a not uncommon dose–response phenomenon also observed in other exposure-disease contexts [49]. Other studies testing for non-linear effects of traffic-related air pollutants on fetal growth have been mixed [23, 50, 51]. Interestingly, we found a similar non-linear dose–response between cigarettes/day and birth weight, an effect also shown by England et al. using both self-reported cigarettes/day as well as urine-cotinine levels to assess exposure [52].

Our results show a negative interaction between PM_2.5_ and SES such that a more pronounced effect of PM_2.5_ was seen in lower SES neighbourhoods (Fig. 3a); however this result could be sensitive to collider bias. We also observed significant interactions between PM_2.5_ and Asian immigration density as well as with PM_2.5_ and living in a rural location (Fig. 3b and c respectively). This suggests that that neighbourhood characteristics can not only influence fetal growth but can also modify exposures either positively or negatively. The biological mechanisms supporting such interactions have been recently reviewed [5], and have been indirectly supported in epidemiological studies that found stronger effects of PM_2.5_ across race, age and SES groups [23, 25, 53]. For example, the observed lower birth weights associated with neighbourhoods with higher densities of continental Asian immigrants is likely due to constitutional birth size differences [37, 38], but the positive interaction with PM_2.5_ may reflect the buffering effect of strong community cohesiveness and beneficial cultural practices [23, 32]. A similar interaction was found by Basu et al. in which births to Asian mothers exhibited smaller birth weight reductions for PM_2.5_ constituents compared to Caucasian births [23]. Currie et al. however did not find any significant interactions between traffic-related carbon monoxide exposure and risk factors such as race, education, or low income [54].

The significant negative interaction between rural address and PM_2.5_ may reflect the underestimation of PM_2.5_ in rural areas by the LUR model [34]. The composition of PM_2.5_, and thus its relative toxicity, is shown to vary spatially depending on its source (e.g., wood smoke vs. traffic-related emissions) and may partially explain the observed rural–urban differences [4, 6]. The significant negative association between season of birth and birth weight could also reflect the increased presence of wood heating and vehicle exhaust in combination with winter stagnation events, but could also reflect a change in diet or increased infection rates [4, 55]. An interaction between season of birth and PM_2.5_ was not statistically significant.

Interactions between PM_2.5_ and maternal-level variables shown to reduce birth weight independently revealed some counter-intuitive results. This included the PM_2.5_ interaction with maternal smoking (cigarettes/day) and with suspected drug/alcohol use where increasing PM_2.5_ levels tempered the negative effect of these risk factors (Fig. 2a and b). This finding was counterintuitive to our original hypothesis and published literature [54], and gave rise to the suspicion of survival bias due to competing risks (i.e., risk behaviours leading to early miscarriage, preterm or stillbirths). Although we were not able to control for fetal loss prior to 20 weeks gestation, survival bias was mitigated by using a near full population sample that included stillbirths, congenital anomalies and preterm births. Furthermore, the positive interaction between maternal smoking and PM_2.5_ was unchanged after the sensitivity analyses; however, the interaction between drug and alcohol use and PM_2.5_ remained positive but was no longer significant (*p* = 0.054). The persistence of this finding leads to a hypothesis that some individual-level exposures may act as a pre-conditioning stress that activates an adaptive response of increased biological resistance to similar stressors [56].

A protective effect of older maternal age against PM_2.5_ exposure was also observed by Basu et al. [23], and may stem from increased nutritional awareness among older women and/or more secure income and support networks thereby reducing potential stress and anxiety [57, 58]. Currie et al. also found significant interactions between traffic-related carbon monoxide exposure and maternal age, but that both younger (< age 19) and older (> age 34) maternal age had greater reductions in birth weight [54]. Gestational diabetes has been shown to be associated with PM_2.5_ and other air pollutants [59, 60]; however, their interaction with respect to birth weight has not been assessed. Our study showed that pregnancies affected by gestational diabetes had significantly higher birth weights as expected but revealed a sharp reduction in birth weight with increasing PM_2.5_. This significant negative interaction between PM_2.5_ and gestational diabetes could be related to excess of systemic or placental oxidative stress and inflammation resulting in restricted fetal growth [15, 61].

While the application of multilevel models in perinatal epidemiology has become more common [62], most have been random-intercept models with very few including a random-slope parameter. Permitting the slope for individual level-1 gestational age to be random can elucidate how its effect on birth weight differs between DAs. For example, the addition of level-1 covariates reduced the random-slope variability from 30.1 to 26.6. This suggests that that these maternal risk factors act through gestational age to influence birth weight and are not distributed homogeneously across DAs. In light of these findings, significant inter-DA variance remained for both the random intercept and slope. In other words, despite explaining a substantial proportion of the between-DA variance in birth weight with both level-1 compositional and level-2 contextual factors, there remained unmeasured DA-level mechanisms acting either directly on fetal growth and/or through gestational age to produce between neighbourhood differences in birth weight.

Spatial analyses were used to examine the wider spatial context within which the DAs are situated and also served as a measure of model specification. The inclusion of the DA-level variables and interactions substantially reduced the spatial autocorrelation in the level-1 and level-2 random-intercept residuals (L1 & L2ri Moran’s I in Table 5). There was very little spatial autocorrelation in the level-2 random-slope parameters (L2rs Moran’s I in Table 5), but it was also reduced in the DA-level models. This suggests that the census DAs perform well in capturing neighbourhood-level processes and that the selected DA-level variables do well to addess the underlying spatial processes acting on birth weight at this neighbourhood-level.

A key component of this research was the use of a land-use regression (LUR) model of air pollution [34]. While the LUR model was independently validated and achieved decent overall results in its predicted estimates, the very nature of our study design ensured some degree of exposure misclassification to our study population. Our analysis was based on maternal place of residence at delivery, and therefore intra-urban commuting and potential inter-urban relocation within the pregnancy period was not accounted for which could affect the results. Time-activity patterns show that pregnant women spend more time at home in the later stages of pregnancy, but mobility patterns may differ by age, parity and SES [63, 64]. Another limitation regarding the PM_2.5_ exposure assessment is that the LUR model is cross-sectional based on 2006 air quality monitoring data, while the study period of our perinatal dataset spans 6 years (2001 to 2006). We therefore assume all pregnancies were exposed to the same levels of PM_2.5_ for their entire pregnancy, regardless of their year of birth, based on their residential DA. While this method prevents the assessment of exposure windows by trimester, spatiotemporal studies of PM_2.5_ have shown little to no difference between trimester-specific and entire pregnancy effects on birth weight [3, 46, 48]. Finally, the mean PM_2.5_ concentrations may be underestimated by the LUR model with less variability and missing several high PM_2.5_ outlier locations in BC compared to compiled monitored data [26]. This could potentially result in an underestimation of our observed association of reduced birth weight with increasing PM_2.5_ levels.

We were unable to control for maternal-level SES, and therefore the neighbourhood-level effect estimates and interactions could reflect individual-level differences. For example, the protective effect of older maternal age buffering the PM_2.5_ effect on birth weight could be due to individual-level SES factors not accounted for in our models such as diet, income or stress. However, studies have found that adjustment for individual-level measures of SES did not significantly change the area-level associations [20, 65]. Maternal education is a variable provided in the BC Perinatal Data Registry, but was only available for 10 % of our population. However, the adjustment for socially-patterned behavioural risk factors such as maternal smoking, suspected drug or alcohol use and low number of prenatal care visits will control for some individual-level SES differences [66].

## Conclusions

This study supports the growing literature of an effect of PM_2.5_ on birth weight and its modification by both maternal and neighbourhood-level factors. Most notably, it shows that lower SES neighbourhoods may be more negatively affected by higher levels of PM_2.5_. We observed both positive and negative interactions between maternal factors and PM_2.5_ that require further scrutiny but may reflect a PM_2.5_-oxidiative stress pathway expressed via either protective pre-conditioning or harmful overload. Targeted municipal-level interventions to reduce PM_2.5_ and improved neighbourhood SES may help improve birth outcomes at the population-level.

## Abbreviations

BC: British Columbia
CSD: census subdivision
DA: dissemination area
ICC: intra-class correlation
L1: level-1
L2: level-2
LHA: local health area
LUR: land use regression
NO_2_: nitrogen dioxide
PCV: proportional change in variance
PM_2.5_: particulate matter less than 2.5 microns in diameter
PSBC: Perinatal Services British Columbia
RI: random intercept
RS: random slope
SES: socio-economic status
SESi: socio-economic status index
